# Improved Performance of GaN-Based Light-Emitting Diodes Grown on Si (111) Substrates with NH_3_ Growth Interruption

**DOI:** 10.3390/mi12040399

**Published:** 2021-04-05

**Authors:** Sang-Jo Kim, Semi Oh, Kwang-Jae Lee, Sohyeon Kim, Kyoung-Kook Kim

**Affiliations:** 1 School of Materials Science and Engineering, Gwangju Institute of Science and Technology, Gwangju 61005, Korea; prokimsj@gmail.com; 2 Department of Electrical Engineering and Computer Science, University of Michigan, Ann Arbor, MI 48109, USA; ohsemi1230@gmail.com; 3 Department of Electrical Engineering, Stanford University, Stanford, CA 94305, USA; kwangjae@stanford.edu; 4 Department of Advanced Convergence Technology, Research Institute of Advanced Convergence Technology, Korea Polytechnic University, 237 Sangidaehak-ro, Siheung-si 15073, Korea; sohyeon.kim@kpu.ac.kr

**Keywords:** AlN buffer layer, NH_3_ growth interruption, strain relaxation, GaN-based LED, low defect density

## Abstract

We demonstrate the highly efficient, GaN-based, multiple-quantum-well light-emitting diodes (LEDs) grown on Si (111) substrates embedded with the AlN buffer layer using NH_3_ growth interruption. Analysis of the materials by the X-ray diffraction omega scan and transmission electron microscopy revealed a remarkable improvement in the crystalline quality of the GaN layer with the AlN buffer layer using NH_3_ growth interruption. This improvement originated from the decreased dislocation densities and coalescence-related defects of the GaN layer that arose from the increased Al migration time. The photoluminescence peak positions and Raman spectra indicate that the internal tensile strain of the GaN layer is effectively relaxed without generating cracks. The LEDs embedded with an AlN buffer layer using NH_3_ growth interruption at 300 mA exhibited 40.9% higher light output power than that of the reference LED embedded with the AlN buffer layer without NH_3_ growth interruption. These high performances are attributed to an increased radiative recombination rate owing to the low defect density and strain relaxation in the GaN epilayer.

## 1. Introduction

Substantial progress in fabricating highly efficient, GaN-based, multiple-quantum-well (MQW) light-emitting diodes (LEDs) has proven these materials useful in a variety of applications, such as micro-displays, automobiles, general lighting, and optoelectronics [1,2,3]. Furthermore, GaN-based epitaxial-layers for LED devices are conventionally fabricated on sapphire and SiC substrates. However, the sapphire substrate has poor thermal conductivity—as low as 25 Wm^−1^K^−1^—that encourages heat dissipation. Therefore, it is a critical issue in high-output LED operation. Additionally, GaN and SiC substrates are expensive and still limitedly used in large-scale LED fabrication than a sapphire substrate [4,5].

The large-scale Si substrate has attracted significant interest in growing GaN-based devices owing to lower cost than the traditional sapphire and SiC substrates [6,7]. Moreover, Si substrate can be easily integrated with electronic and optical devices [8].

However, growing a high-quality GaN epitaxial layer on a Si (111) substrate presents several key challenges. First, when the GaN layer is grown directly on the Si substrate, the Si surface easily reacts with NH_3_ to form the SiN_x_, which the GaN layer cannot grow. Subsequently, the Si substrate reacts with Ga to promote melt-back etching of Ga-Si eutectic alloys [9]. Second, the large lattice mismatch (~17%) between GaN and Si (111) causes a high dislocation density in the GaN layer leading to lower LED performance [10]. Third, the difference in thermal expansion coefficients (~56%) between Si and GaN introduces large tensile stress in the GaN layer during the cooling process from the high growth temperature, which causes the wafer bowing and cracks generation [11].

Therefore, many researchers have introduced various methods to reduce threading dislocation, stress mitigation, and remove cracks, such as epitaxial lateral overgrowth, nanoporous GaN layers, graded AlGaN interlayers, and Al(Ga)N/GaN superlattices [12,13,14,15]. Particularly, the AlN layer, which acts as a bottom buffer layer on the Si substrate, significantly affects crystalline quality and stress management of the GaN layer.

AlN layers with rough surfaces and poor crystalline qualities lead to GaN layers with poor crystalline quality. Therefore, many studies have revealed that high-quality AlN buffer layers minimize crystal misorientations and dislocation density in the GaN layer. Krost et al. developed a low-temperature (LT)-AlN layer—that is, a novel method to reduce stress [16].

Comparatively, high-temperature (HT)-AlN layers yield reduced dislocation densities and promote the relaxation of compressive strain due to an increased Al migration length. In addition, many studies are also investigated the high-quality GaN layer grown on a Si substrate using the HT- and LT-AlN growth process [17,18]. Moreover, Hirayama et al. used an AlN buffer layer grown with pulsed NH_3_ flow on a sapphire substrate [19], which is an effective way to enhance the lateral migration of Al atoms and produce a smooth epitaxial surface.

However, there have been no reports on the effects of ammonia (NH_3_) growth interruption for the AlN layer in GaN-based LEDs grown on Si (111) substrates.

We demonstrate that AlN layers prepared with NH_3_ growth interruption to be served as a buffer layer improve the crystalline quality and strain relaxation in GaN-based LEDs grown on Si (111) substrates. The Al migration time of the AlN was controlled by NH_3_ pulse timing.

The optical output power of LEDs grown using NH_3_ growth interruption was 40.9% greater than that of the reference LED embedded with the AlN buffer layer without NH_3_ growth interruption (injection current of 300 mA). Such remarkable improvements of optical output power are predominantly attributed to reduced dislocation densities and strain relaxation, which originated from an increased Al migration in the AlN buffer layer.

## 2. Materials and Methods

**Figure 1:**
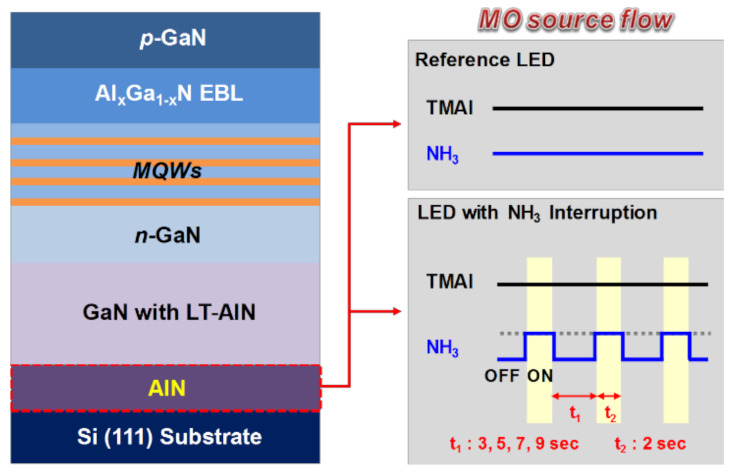
Schematics of light-emitting diodes (LEDs), including the sequence of all growth steps prepared with and without the NH_3_ growth interruption method.

InGaN/GaN MQW LEDs were grown on Si (111) substrates using metal-organic chemical vapor deposition. Trimethylaluminum (TMAl) and NH_3_ were used as the Al and N sources, respectively, and high-purity hydrogen was employed as the carrier gas. Si (111) substrates were first annealed at 1050 °C for 10 min to remove the native oxide. The substrates were subsequently passivated by TMAl with a pre-dose time of 10 s to prevent Si melt-back etching. A 200 nm-thick AlN layer was subsequently deposited at 1050 °C as a reference. Comparatively, for samples prepared by NH_3_ growth interruption, TMAl was constantly introduced into the chamber while NH_3_ was injected into the reactor following a specified pulsed regimen. Specifically, the halted (*t*_1_) NH_3_ flow time was changed: 3 s for sample A, 5 s for sample B, 7 s for sample C, and 9 s for sample D, as schematically shown in Figure 1.

After that, to prevent crack formation, two pairs of 12 nm-thick low-temperature AlN layers and a 1.2 µm-thick undoped GaN layer were deposited.

Furthermore, a 1.5 µm-thick *n*-type GaN layer (*n* = 6 × 10^18^/cm^3^), six periods of 2.3 nm-thick In_0.18_Ga_0.82_N layer, and 7.7 nm-thick GaN-based MQWs layers were subsequently grown. The 15 nm-thick Al_0.15_Ga_0.85_N electron blocking layer (EBL) and the 200 nm-thick *p*-type GaN layer ((*n* = 5 × 10^19^/cm^3^) were finally deposited.

To fabricate the n-electrode, the epilayers were partially etched until the *n*-type GaN layer was exposed. The 200 nm-thick ITO layer was deposited using an electron-beam evaporator on the remaining parts of the p-type GaN layer and annealed at 600 °C in O_2_ atmosphere for 1 min using the rapid thermal annealing. The Ti/Al (50/200 nm) layers were deposited as an n-electrode. Finally, the Cr/Al (30/200 nm) layers were deposited on the *p*- and *n*-electrodes and annealed at 300 °C for 1 min.

## 3. Results and Discussion

### 3.1. Epitaxial Characteristics

**Figure 2:**
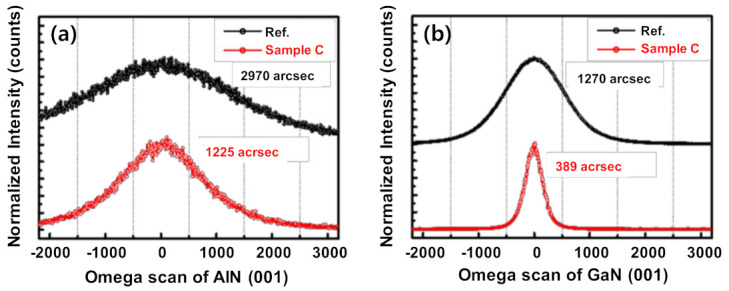
X-ray diffraction (XRD) Omega scan of reference and sample C (**a**) AlN (001) and (**b**) GaN (001).

**Figure 3:**
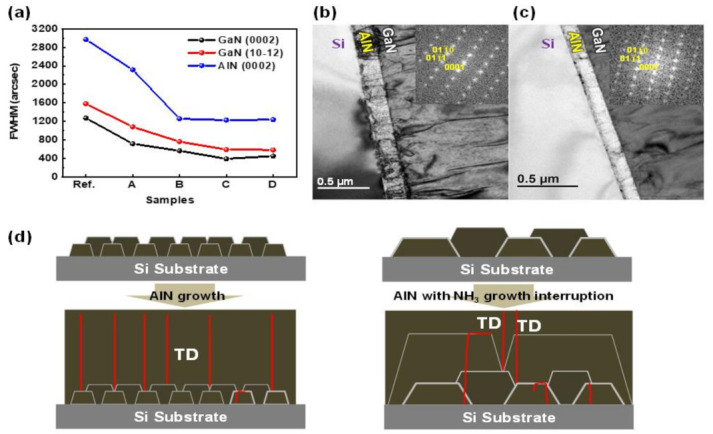
(**a**) The full-width-at-half-maximum (FWHM) values of the GaN (0002), GaN (10–12), and AlN (0002) reflections for all the samples. TEM images of (**b**) a reference sample and (**c**) a sample C. (**d**) Schematic growth mechanism of AlN with and without the NH_3_ growth interruption.

The effect of NH_3_ growth interruption on the crystal quality of GaN was explored using X-ray diffraction (XRD) (PANalytical X’Pert PRO, Almelo, Netherlands) (reference sample and Sample C), as shown in Figure 2a,b. In addition, all samples of full-width-at-half-maximum (FWHM) values of the GaN (0002), GaN (10–12), and AlN (0002) reflections are plotted in Figure 3a.

The full-width-at-half-maximum (FWHM) of the (0002) and (10–12) reflections of GaN and AlN layers typically indicate imperfections in the crystal showing the densities of the screw (*D_s_*) and edge (*D_e_*) dislocations in the epitaxial layer, respectively. The parameters *D_s_* and *D_e_* can be obtained using the following equations [20,21]:

(1) $$D_{S}=\beta_{\left(0002\right)}^{2}/4.35b_{c}^{2}$$

(2) $$D_{e}=\beta_{\left(10-12\right)}^{2}/4.35b_{a}^{2}$$

where *β*^2^_(0002)_ and *β*^2^_(10–12)_ denote the FWHM of the (0002) and (10–12) reflections, respectively; *b_c_* and *b_a_* represent the Burgers vector lengths of the *c*- and *a*-axial lattice constant, respectively. The FWHM values of the (0002) and (10–12) reflections of GaN were 714 and 1079 arcsec for sample A, 563.4 and 760.3 arcsec for sample B, and 389 and 589.3 arcsec for sample C, respectively. These FWHM values are much lower than the corresponding FWHM values (1270 and 1580 arcsec) of a reference sample. The corresponding *D*_s_ and *D*_e_ are tabulated in Table 1. These results contain similar values to the other papers mentioned in introduction of HT-AlN, and LT-AlN growth for high-quality GaN layer grown on a Si substrate [17,18].

Al adatoms have a high sticking coefficient and short migration time on Si substrates; therefore, they have a significantly low probability of moving from their point of impact. This gives rise to many nucleation sites for growth with a high density of extended defects, such as dislocations and grain boundaries [22]. Moreover, growth interruption by halting NH_3_ gas flow provides Al adatoms with sufficient residence time to incorporate the most energetic favorable lattice sites on the Si substrate. It promotes a large number of small nucleation sites, which further minimizes coalescence-related defects. However, significantly long migration time for sample D promotes AlN layers with lower crystalline quality and increased dislocation densities compared to those of sample C due to the deteriorating surface roughness, as shown in Table 1 [23,24,25].

We analyzed transmission electron microscopy (TEM) to deeply investigate the crystalline quality of AlN layers produced using NH_3_ growth interruption. Figure 3b,c show the cross-sectional bright-field TEM images of the reference sample and sample C, respectively, which were acquired near the GaN [0001] zone axis. Dislocations were clearly observed at the interface of the AlN and Si in the reference material; however, they are remarkably reduced in sample C because the longer Al migration time (as shown in Figure 3d) decreases coalescence related defects and dislocation densities.

**Figure 4:**
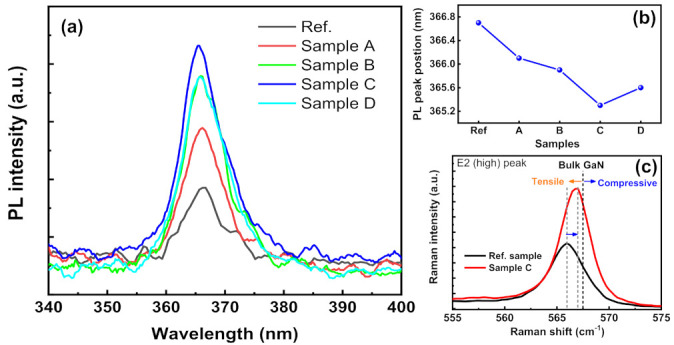
(**a**) Photoluminescence (PL) intensity and (**b**) PL peak position of all the studied samples. (**c**) Raman spectra of the reference sample and sample C.

The impact of the NH_3_ growth interruption process on the residual stress in the GaN epilayer was quantified by photoluminescence (PL) and Raman measurements, as shown in Figure 4a,b, respectively. The PL intensities of samples prepared by the NH_3_ growth interruption process increased with Al migration time (*t*_1_), indicating higher GaN crystal quality than the other samples. However, the PL intensity decreased with longer Al migration times. Moreover, the PL peak position of a sample C blue-shifted from 366.7 nm to 365.3 nm and subsequently red-shifted to 365.6 nm in sample D, indicating that the residual tensile strain in sample C is smaller than that in the other samples. Figure 4c shows the Raman spectra acquired from the reference sample and sample C, specifically using a 514 nm light with the excitation power of 2.4 mW.

The *E*_2_ (high) vibrational mode is highly sensitive to strain. Therefore, it is widely used to quantify the stress in GaN epilayers [25]. The wavenumber of the *E*_2_ (high) mode of the reference sample and sample C was 566.03 cm^−1^ and 567.18 cm^−1^, respectively. Both the values were red-shifted from the *E*_2_ (high) wavenumber from a standard free-standing bulk GaN (567.5 cm^−1^), proving that the GaN epilayers in the reference and sample C were under tensile stress [26]. Shifts of the *E*_2_ (high) phonon peak are related to the relaxation of residual strain; it can be calculated using the following equation [27]:

(3) $$\Delta \omega_{\gamma }-\omega_{0}=K_{\gamma }\cdot \sigma_{xx}$$

where *ω_γ_* and *ω*_0_ represent the Raman wavenumbers of the *E*_2_ (high) phonon peak of sample C and reference sample, respectively. A proportionality factor *K_γ_* (4.2 cm^−1^ GPa^−1^) originates from hexagonal GaN [28]. The *E*_2_ (high) phonon peak of sample C was blue-shifted by 1.15 cm^−1^ from that of the reference sample, corresponding to the impressive different relaxation of tensile stress (*σ_xx_*) (0.274 GPa). The relaxation of tensile strain in GaN layer for sample C causes the shrinking the lattice constant, which shifts the PL peak position to higher energy [29]. The reduced tensile stress in sample C arises predominantly from the coalescence of large-sized grains due to the NH_3_ growth interruption method [30,31].

### 3.2. Device Characteristics

**Figure 5:**
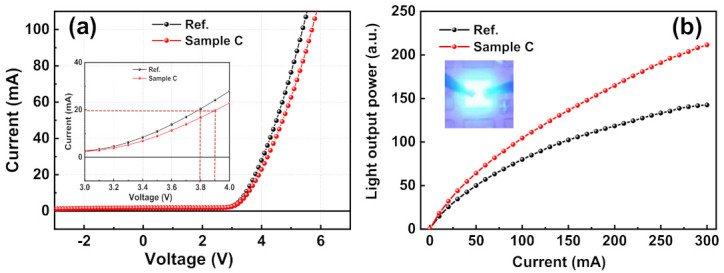
(**a**) Current-voltage (I-V) characteristics and (**b**) optical output power of the reference sample and a sample C.

Figure 5 shows the current-voltage (I-V) characteristics and light output power of the reference sample and sample C. Figure 5a reveals that the forward voltage of sample C at an injection current of 20 mA was 3.76 V, which is lower than that of the reference sample (3.91 V) under the same condition.

Furthermore, the series resistances estimated from the I-V curves of the reference sample and sample C were 37.2 ohm and 31.6 ohm, respectively. These values indicate that decreasing the density of defects, such as threading dislocations by NH_3_ growth interruption method, improves the crystal quality of GaN epi-layer; moreover, it results in a decreased series resistance and forward voltage. Figure 5b shows that the light output power of sample C at an injection current of 300 mA is 40.9% greater than that of the reference sample; it is attributed to enhanced radiative recombination due to the reduced dislocation densities and relaxation of internal tensile strain. We could not measure the light output power above 300 mA by the limitation of measurement system. However, normally, as the injection current is increased, the carrier overflow and Auger recombination are also increased. Therefore, at high current, the portion of increasing light output power will be decreased [32,33].

## 4. Conclusions

We demonstrated the NH_3_ growth-interruption process to prepare the high-performance InGaN/GaN MQW LEDs grown on Si (111) substrates. The XRD results revealed low FWHM values for GaN grown on AlN layers using the NH_3_ growth interruption method; it established substantially improved crystal quality compared to the reference LED embedded with the AlN buffer layer without NH_3_ growth interruption.

Improved crystalline quality is further corroborated by comparative TEM analyses, which is attributed to the effectively reduced dislocation densities and coalescence by longer Al migration times. A lower forward voltage of 3.76 V was observed at an injection current of 20 mA for the LED fabricated by the NH_3_ growth interruption method; however, the reference LED had a forward voltage of 3.91 V. The optical output power of the LED prepared using the NH_3_ growth interruption was 40.9% greater than that of the reference LED.

Such enhanced optical output is attributed to increased radiative recombination rates due to the decreased dislocation densities and relaxation of internal tensile strain, which arise from the longer Al migration time on the Si (111) substrate. These results demonstrate that the NH_3_ growth interruption method is an important technique for growing high-performance LEDs grown on Si (111) substrate. Therefore, these results ultimately represent a step towards realizing high-efficiency and high-power LEDs.

## Figures and Tables

**Table 1:** The full-width-at-half-maximum (FWHM) values of the X-ray diffraction (XRD) rocking curve and calculated dislocation densities for all the samples.

Sample	*t*_1_/*t*_2_	XRD FWHM (arcsec)			Dislocation Density (×10^9^ cm^−^^2^)		
		GaN (001)	GaN (102)	AlN (001)	*D_s_* (GaN)	*D_e_* (GaN)	*D_s_* (AlN)
Ref.	*t_1_*	1270	1580	2970	13.8	56	81.7
Sample A	3/2	714	1079	2316	4.36	26.3	49.7
Sample B	5/2	563	760	1255	2.71	13	14.6
Sample C	7/2	389	589	1225	1.29	7.85	13.9
Sample D	9/2	448	581	1237	1.71	7.63	14.2
